# *Roisinitermesebogoensis* gen. & sp. n., an outstanding drywood termite with snapping soldiers from Cameroon (Isoptera, Kalotermitidae)

**DOI:** 10.3897/zookeys.787.28195

**Published:** 2018-08-02

**Authors:** Rudolf H. Scheffrahn, Thomas Bourguignon, Pierre Dieudonné Akama, David Sillam-Dussès, Jan Šobotník

**Affiliations:** 1 Fort Lauderdale Research and Education Center, Institute for Food and Agricultural Sciences, 3205 College Avenue, Davie, Florida 33314, USA Institute for Food and Agricultural Sciences Davie United States of America; 2 Okinawa Institute of Science & Technology Graduate University, 1919-1 Tancha, Onna-son, Okinawa 904-0495, Japan Czech University of Life Sciences Prague Czech Republic; 3 Faculty of Forestry and Wood Sciences, Czech University of Life Sciences, Prague, Czech Republic Okinawa Institute of Science & Technology Graduate University Tancha Japan; 4 Département des sciences biologiques, Ecole normale supérieure, Université de Yaoundé I, BP 47 Yaoundé, Cameroon Université de Yaoundé I Yaoundé Cameroon; 5 University Paris 13 - Sorbonne Paris Cité, LEEC, EA4443, Villetaneuse, France University Paris Paris France; 6 IRD – Sorbonne Universités, iEES-Paris, Bondy, France Sorbonne Universités Paris France

**Keywords:** Ethiopian Region, mandibles, ocellus, taxonomy

## Abstract

Termites have developed a wide array of defensive mechanisms. One of them is the mandibulate soldier caste that crushes or pierces their enemies. However, in several lineages of Termitinae, soldiers have long and slender mandibles that cannot bite but, instead, snap and deliver powerful strikes to their opponents. Here, we use morphological and molecular evidence to describe *Roisinitermesebogoensis* Scheffrahn, **gen. & sp. n.** from near Mbalmayo, Cameroon. Soldiers of *R.ebogoensis* are unique among all other kalotermitid soldiers in that they possess snapping mandibles. The imago of *R.ebogoensis* is also easily distinguished from all other Kalotermitidae by the lack of ocelli. Our study reveals a new case of parallel evolution of snapping mandibles in termites, a complex apparatus responsible of one of the fastest biological acceleration rates measured to date.

## Introduction

Termites are extremely abundant (Martius 1994, Eggleton et al. 1996) and colonies may contain millions of individuals attracting a wide variety of predators (Deligne et al. 1981). Additionally, termites experience strong intra- and inter-specific competition (Levings and Adams 1984, Thorne and Haverty 1991). To combat against the plethora of agonistic opponents, termites have developed a rich array of defensive strategies. The most important defenses are expressed in the soldier caste that is ancestral to all extent termites (Roisin 2000).

Soldiers are specialized sterile colony defenders possessing exaggerated morphology of the head and mandibles (Prestwich 1984). One of their most intriguing defenses is exemplified by long and slender snapping mandibles (Deligne et al. 1981). The snapping mandibles are paired with muscles to store potential energy which, when released, delivers a powerful strike producing one of the fastest accelerations known among animals (Seid et al. 2008). All termite species with snapping soldiers described so far belong to the Termitinae (Bourguignon et al. 2017), suggesting that snapping soldiers evolved several times independently within this subfamily. Alternatively, soldiers with snapping mandibles might have evolved once, and independently reverted to a biting strategy in several lineages.

The monophyletic family Kalotermitidae (Inward et al. 2007) constitutes almost half of all “lower termite” genera and species (Krishna et al. 2013) and has fossil records to the mid-Cretaceous (Engel et al. 2009). Kalotermitids live entirely in wood as “one-piece” nesters (Abe 1987) which facilitates transoceanic dispersal (Scheffrahn and Postle 2013). Kalotermitids occur in all ecozones and numerous genera have vast distributions (e.g. *Calcaritermes*, *Cryptotermes*, *Glyptotermes*, *Kalotermes*, *Marginitermes*, *Neotermes*, and *Procryptotermes*). A few species of *Cryptotermes* (Scheffrahn et al. 2009) and *Incisitermes* (James et al. 2013, Yasuda et al. 2003) have also been dispersed by human activity. A few species are major pests of dry wood (Su and Scheffrahn 2000) or minor pests of tree crops (Constantino 2002).

The monumental revision of the Kalotermitidae by Krishna (1961) provided the morphological diagnoses for all extant genera with the exception of the recently described *Longicaputermes* (Ghesini et al. 2014). Aside from *Longicaputermes*, all new kalotermitid species described after Krishna’s 1961 revision, ca. 115, have been assigned to one of the 21 genera he recognized. The soldier caste of several genera has unmistakable characters: e.g., the scooped out frons of *Eucryptotermes*, the massive third antennal article of *Marginitermes*, the large ovoid head of *Pterotermes*, or the spur on the fore tibia of *Calcaritermes*. We herein describe a new genus and species of Kalotermitidae, *Roisinitermesebogoensis*, which possesses equally unmistakable soldiers. The soldier of *R.ebogoensis* is the first outside the Termitinae to have snapping mandibles.

## Material and methods

### Illustrations and measurements

Images of individuals were taken as multi-layer montages using a Leica M205C stereomicroscope with a Leica DFC 425 module run with Leica Application Suite software version 3. Preserved specimens, stored in 85% ethanol, were positioned in a transparent petri dish filled with Purell hand sanitizer (70% EtOH). Measurements (Tables 1–2) were obtained using an Olympus SZH stereomicroscope fitted with an ocular micrometer. A field photograph of live specimens placed in a small paper-lined Petri dish was taken with a Canon EOS 5DS R combined with a Canon EF 100mm f/2.8L Macro IS USM lens. Morphological terminology follows that of Krishna (1961).

### Phylogenetic analyses

DNA was extracted from five individuals of *R.ebogoensis*, after removal of the digestive tract. The full mitochondrial genome was amplified with TaKaRa LA Taq in two long PCR reactions using primers specifically designed for termites (Bourguignon et al. 2016). Long PCR fragments were pooled in equimolar concentration, and 75-bp paired-end reads were obtained using Illumina MiSeq. We subsampled a total of 10,000 reads and assembled the full mitochondrial genome with SPAdes, under default parameters (Bankevich et al. 2012). The total coverage of the assembly was 82-fold.

We used the mitochondrial genomes of ten species of Kalotermitidae, including one sample of *Roisinitermesebogoensis* sequenced in this study. We used four non-Kalotermitidae termite species as outgroups to root the tree: *Zootermopsisangusticollis*, *Hodotermopsissjostedti*, *Coptotermessjostedti*, and *Termitogetonplanus*. All mitochondrial genomes, except that of *R.ebogoensis*, have been published recently (Suppl. material 1: Table S1). Each gene of the mitochondrial genome was aligned separately using MAFFT v7.300b with the option “--maxiterate 1000 --globalpair” for higher accuracy. For protein-coding genes, we first aligned genes as protein, then converted protein sequence alignments into the corresponding codon alignments using PAL2NAL (Suyama et al. 2006). The 22 tRNAs and the two ribosomal RNAs were aligned as DNA. The resulting alignments were concatenated with FASTconCAT v1 (Kück and Meusemann 2010). Alignments were separated in five partitions: one for each codon position of the protein-coding genes, one for the combined ribosomal RNA genes, and one for the combined tRNA genes.

We reconstructed phylogenetic trees using Maximum Likelihood and Bayesian approaches. We ran the analyses twice, once with the third codon position included, and once without third codon position. The Bayesian phylogenies were implemented in MrBayes 3.2 (Ronquist et al. 2012) with unlinked partitions, each of four chains (three hot and one cold). The chain length was of two million generations with sampling every 2000 generations. 800,000 generations were discarded as burnin, to ensure that the chain reached convergence, as determined by Tracer 1.5 (Rambaut and Drummond 2007). We ran two replicates of each analysis to ensure consistency of the results. For each partition of the data, we assigned an independent Generalized Time Reversible model with gamma-distributed rate variation across sites and a proportion of invariable sites (GTR + G +I). The reconstruction of Maximum Likelihood phylogenies was carried out with RAxML (Stamatakis et al. 2008). We used the GTRGAMMA model of rate heterogeneity across sites. Node support was estimated using 1000 bootstrap replicates.

## Results

### Phylogenetic analysis

**Figure 1.**
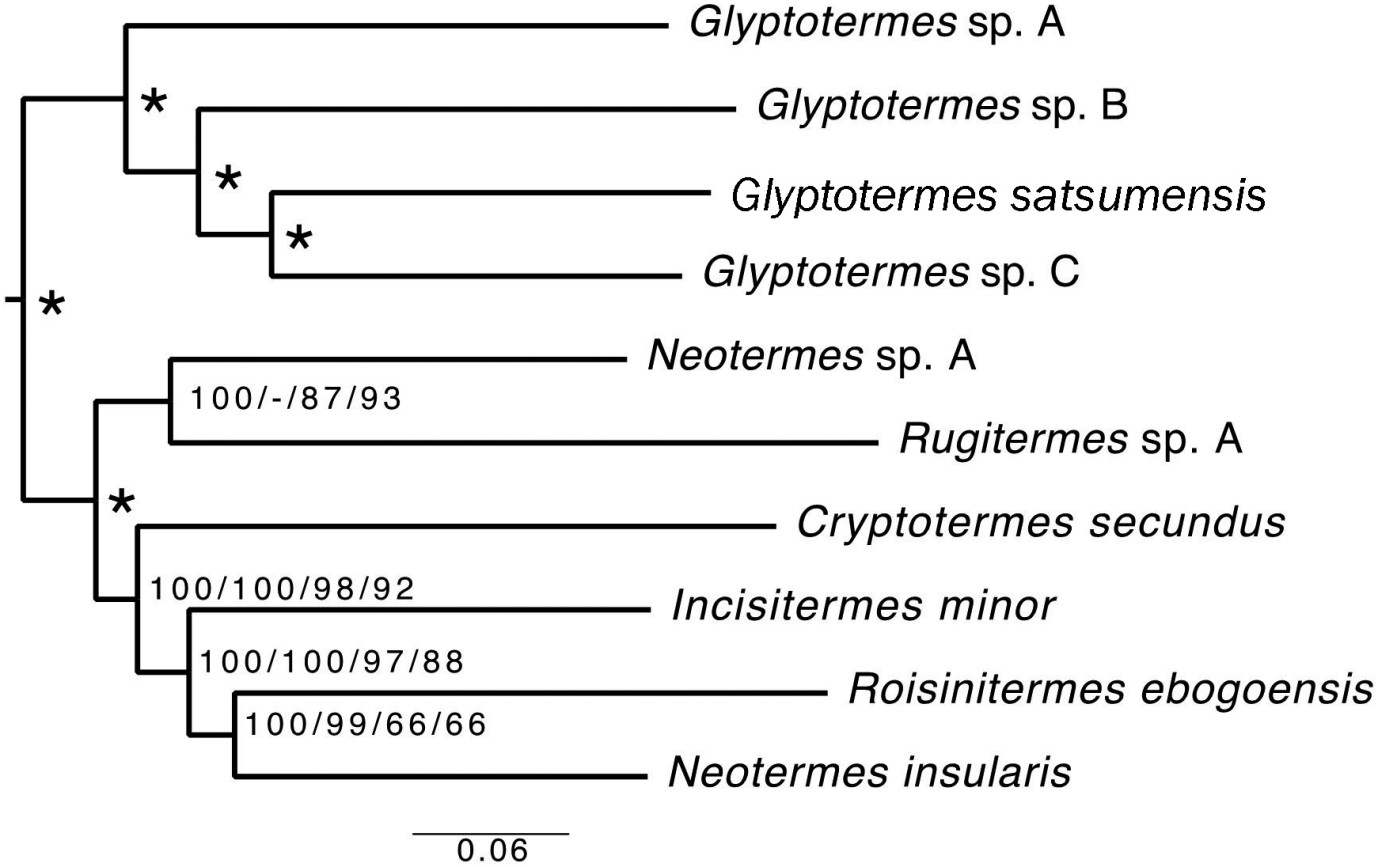
Phylogenetic tree of Kalotermitidae based on full mitochondrial genomes. The tree depicted was reconstructed with RAxML using the data matrix without third codon position. Node labels are the Maximum Likelihood bootstrap supports and the Bayesian posterior probabilities in the following order, from left to right: posterior probability of the analysis with third codon position included, posterior probability of the analysis without third codon position, bootstrap support of the analysis with third codon position included, bootstrap support of the analysis without third codon position, *indicates 100% bootstrap support and 1.0 posterior probability for all four analyses.

Our phylogenetic analyses supported the monophyly of Kalotermitidae (Figure 1). The four analyses yielded identical tree topologies, with one exception: in the Bayesian analysis without third codon position *Rugitermes* was the sister group of *Neotermes* + *Cryptotermes* + *Incisitermes* + *Roisinitermes*, while in the other three analyses *Rugitermes* + *Neotermes* sp. A formed the sister group of *Neotermesinsularis* + *Cryptotermes* + *Incisitermes* + *Roisinitermes*. *Roisinitermes* was consistently placed next to *N.insularis*.

### Systematics

#### Roisinitermes

Taxon classificationAnimaliaIsopteraKalotermitidae

Scheffrahn gen. n.

http://zoobank.org/9AE40F98-CA9E-45AC-849E-A034F19E8DAE

##### Type-species.

*Roisinitermesebogoensis* Scheffrahn sp. n.

##### Winged Imago.

**Figure 2.**
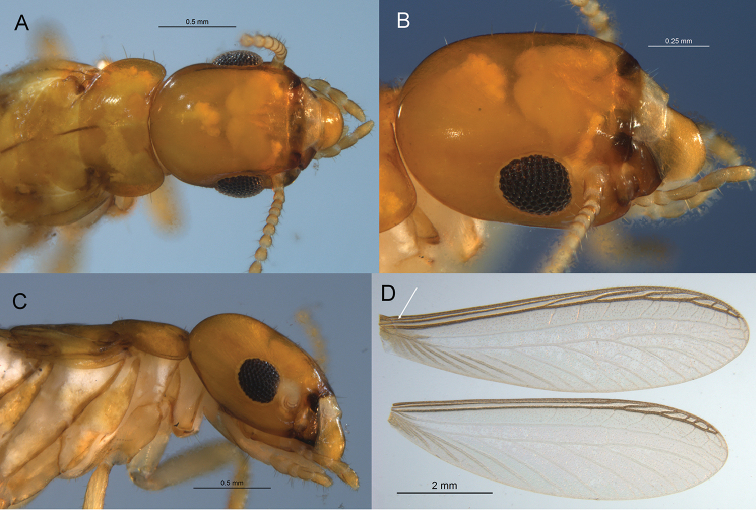
Imago of *Roisinitermesebogoensis* gen. & sp. n. **A** Dorsal view of head and thorax **B** Oblique view of head **C** Lateral view of head and thorax **D** Right forewing (arrow on subcosta) and right hind wing.

**Figure 3.**
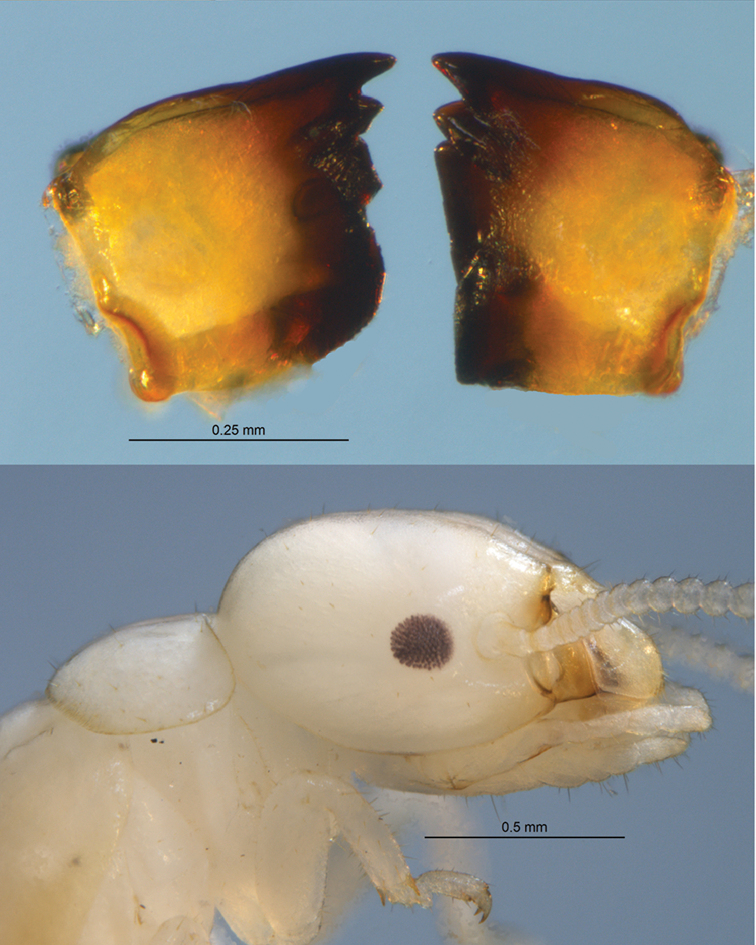
Brachypterous nymph of *Roisinitermesebogoensis* gen. & sp. n. Top: Dorsal view of mandibles. Bottom: lateral view of head and thorax.

Ocelli not visible either by pigmentation or cuticular protrusion (Figure 2A–C). Fore wing with unsclerotized media and cubitus arising from a common vein distal from scale suture; radial sector with 5–6 anterior branches; subcosta very close and difficult to discern from costal margin (Figure 2D). Hind wing with radial sector and cubitus arising from a common vein distal to suture. Tibial spurs 3:3:3; tarsi without arolia. The left imago/nymph mandible with anterior margin of their marginal tooth ca. 1.5 times longer than length of the posterior margin of the first plus second marginal tooth; right mandible with posterior margin of second marginal tooth 1.4 times as long as molar plate (Figure 3).

##### Diagnosis.

The lack of visible ocelli is unique among all other Kalotermitidae. In Krishna’s 1961 generic key, *Roisinitermes* would lead to couplet 2 (*Epicalotermes*).

##### Soldier.

**Figure 4.**
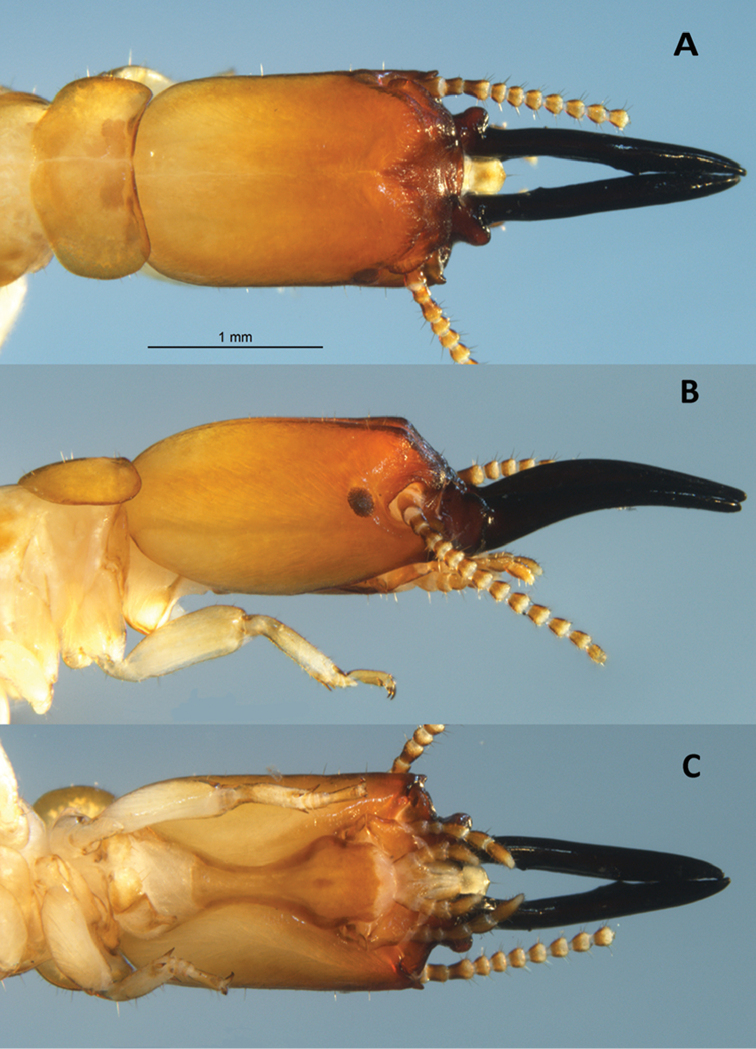
Soldier (holotype) of *Roisinitermesebogoensis* gen. & sp. n. Dorsal (**A**), lateral (**B**), and ventral (**C**) views of head and pronotum.

**Figure 5.**
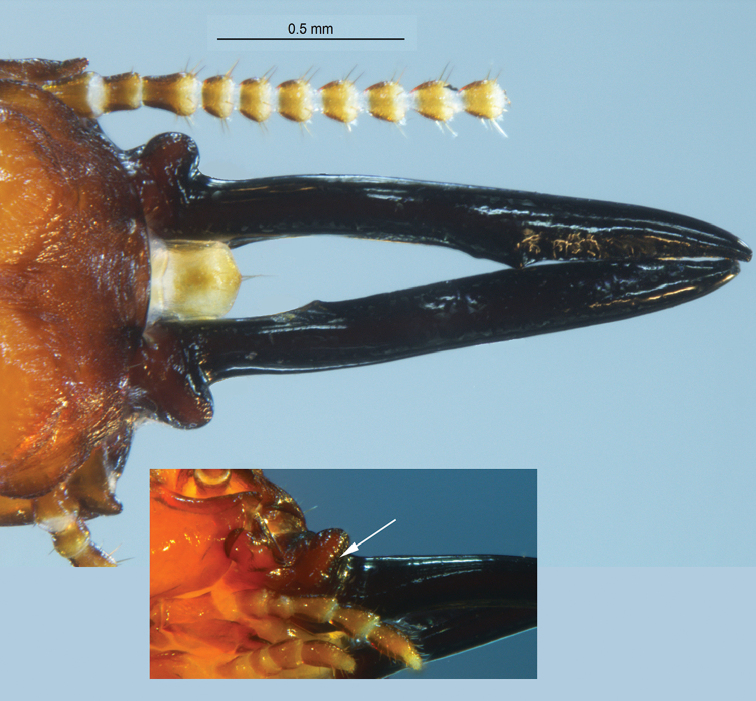
Dorsal view of frons and mandibles of *Roisinitermesebogoensis* gen. n. sp. n. Inset: oblique ventral view of columnar hump (arrow).

Monomorphic (Figs 4, 5). Eye spots prominent; large, dark brown. Frons bilobed in dorsal view, crested with rugose longitudinal wrinkles, rugosity below frons oriented longitudinally. Small horn-like projection at terminus of ventral genae. Mandibles sticklike; downward arching in lateral view. Dentition very weak; basal humps project sharply.

##### Diagnosis.

Stick-like mandibles unique among all other kalotermitid soldiers. In Krishna’s 1961 key, *Roisinitermes* leads to couplet 17 (*Allotermes*). In dorsal view, the mandibular blades of *Allotermes*, especially *A.denticulatus*Krishna 1962, somewhat resemble those of *Roisinitermes* as those of the former are long, rather narrow and with rudimentary dentition. In lateral view, however, the *Roisinititermes* mandibles differ from all other kalotermitids with projecting mandibles in that the *Roisinitermes* mandibles arch downward. Although the *Roisinitermes* imago venation and dentition is very similar to those of *Epicalotermes*, the soldier of *Roisinitermes* shares no major characters with the *Epicalotermes* soldier.

##### Etymology.

The genus is named in honor of Dr. Yves Roisin for his many contributions to the study of termites.

#### Roisinitermes ebogoensis

Taxon classificationAnimaliaIsopteraKalotermitidae

Scheffrahn sp. n.

http://zoobank.org/129573FB-E5DE-4673-9E1B-EF062D413FEB

##### Material examined.

***Holotype*.** Soldier from colony UF no. AFR3327. CAMEROON: Ebogo II, (+3.37723N, +11.46135E), 647 m elev., 18FEB18, col. Raphael Onana, AFR3327 ca. 500 alates, 50 soldiers, and many pseudergates, nymphs, larvae, and eggs. ***Paratypes***. CAMEROON, Ebogo II (+3.38273N, +11.46190E), 664 m elev., 10DEC2016, col. Jan Šobotník and collaborators, AFR2982 4 soldiers (1 damaged), one female dealate, and 46 brachypterous nymphs.

##### Diagnosis.

See generic diagnosis above.

##### Description.

***Winged Imago*** (Figure 2, Table 1) Head and pronotum light brownish orange; eye ovoid, anterior margin truncate abdominal tergites lighter, concolorous with legs and labrum; postclypeus nearly hyaline. Compound eyes black, of medium size and protrusion; ellipsoid but truncated near antennal socket, composed of approximately 85 facets. Ocelli not visible either by pigmentation or cuticular protrusion. Antennae with more than nine articles; formula 1>2=3=4<5. Pronotum width twice that of median length; several long and shorter setae project from lateral margins. Fore wing scale with basal origins of all major veins; wing membrane covered with papillae. Tibial spurs 3:3:3; tarsi without arolia.

**Table 1.** Measurements (mm) of *Roisinitermesebogoensis* alates from a single colony.

	Males (n=6)			Females (n=6)		
Measurement	max	min	mean	max	min	mean
Head max. width	1.05	0.95	1.00	1.05	1.00	1.03
Pronotum max. width	1.00	0.89	0.96	1.05	0.93	1.01
No. antennal articles	15	14	14.67	17.00	14.00	15.17
Max diam. eye	0.40	0.32	0.36	0.39	0.35	0.37
Body length with wings	9.63	8.63	9.10	9.88	9.50	9.65
Fore wing length (suture to tip)	7.50	6.80	7.20	7.80	7.20	7.43

**Figure 6.**
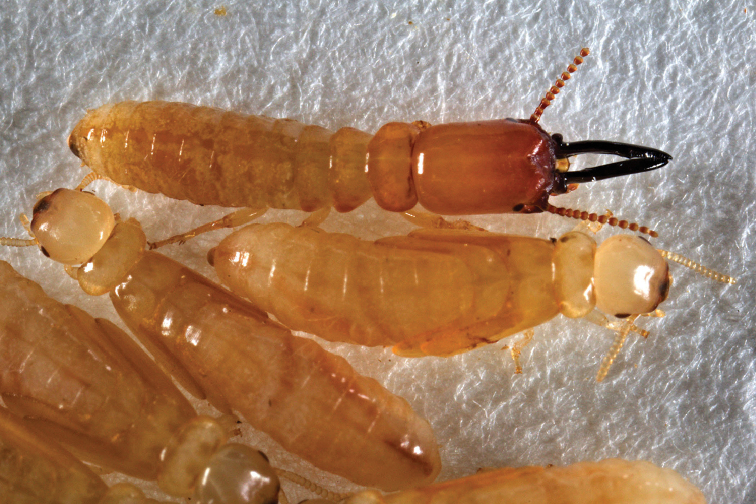
Live habitus of soldier and brachypterous nymphs of *Roisinitermesebogoensis* gen.et sp. n.

***Soldier*** (Figs 4–6; Table 2) Monomorphic. In dorsal view, head capsule yellowish orange in posterior grading to orange in middle and reddish brown from frons to anteclypeus. Three proximal antennal articles sepia brown; distal articles light brown. Post clypeus and labrum yellowish with brown highlights. Eye spots prominent; large, dark brown, elliptical; formed from a mass of discrete ommatidia. Pronotum concolorous with posterior head capsule. Head capsule in dorsal view, subrectangular; lateral margins nearly parallel, length 1.5 times width. Posterior corners of head evenly rounded; posterior margin rectate. In lateral and oblique view, head capsule almost cylindrical with only slight dorso-ventral compression; frons bilobed in dorsal view, crested with rugose longitudinal stripes, rugosity lateral below frons to mandibles. In lateral view, frons sloping from vertex ~45°; mandibles bow upward to form a 15° arch. Setae short and sparse on pronotum and head capsule. Periantennal carina rugose, in dorsal view partially eclipsing the first antennal article. Small horn-like projection at terminus of ventral genae. Mandibles stick-like; long, blade narrower in middle than distal third, dentition very weak; left mandible with faint equilateral tooth approx. three fifths from base, serrations along blade from tooth to tip. Right mandible with single tooth approx. one third distance from base; blade narrowest before tooth; after tooth blade widens slightly and then gradually narrows at tip. In dorsal view, basal humps project sharply as rugose hemispheres. In lateral view, humps are columnar and equal in height to that of the mandibles. Anteclypeus shallowly incised in middle; labrum linguiform with gradual point; 4–5 long terminal setae. Antennae with 12–13 articles, third antennal article subclavate, barely shorter than fourth and fifth combined. Pronotum collar-shaped; much wider than long. Anterior margin weakly concave; lateral margins weakly convex, posterior margin forming 25° angle with incised middle. Femora moderately inflated, tibial spurs 3:3:3. Habitus as in Figure 6.

**Table 2.** Measurements of *Roisinitermesebogoensis* soldier (n=17 from two colonies).

Measurement	Max	Min	Mean
Head length to lateral mandible base	1.92	1.60	1.79
Head width, maximum	1.28	1.18	1.22
Head height with gula, max.	1.08	0.92	1.00
Pronotum length	0.70	0.56	0.65
Pronotum width	1.18	1.05	1.13
No. antennal articles	14	10	12.70
Left mandible width @ basal humps	0.35	0.21	0.26
Left mandible width @ middle	0.18	0.16	0.17
Max. diam. eye	0.26	0.18	0.21
Length left mandible from condyle (ventral)	1.78	1.46	1.66

***Brachypterous nymph*** (Fig. 3, Table 3) Body hyaline. Head, thorax, and abdomen similar in shape and pilosity of imago. Compound eyes with approx. 85 dark facets; both eyes and facets smaller than imago. Antennae with 15 articles; formula 1>2>3=4=5. Left mandible with anterior margin of marginal tooth 1.5 times longer than length of the posterior margin of the first plus second marginal tooth. Right mandible with posterior margin of second marginal tooth 1.4 times as long as molar plate.

##### Biology and distribution.

The type colony of *R.ebogoensis* was collected in the forest on an island in the Nyong River near the Ebogo II village. The colony lived in a relatively thin (3 cm) and long (over 3 m) broad-leaf tree branch suspended from the canopy approximately 2 m above the ground. The colony contained roughly 2,000 members. A second colony of *R.ebogoensis* was collected in a nearly pristine rain forest near the village of Ebogo II. The colony was taken from a dead liana branch (ca. 15 mm diam.) hanging from the canopy at a height of approx. 1 m above the ground. Liana stems have been generally overlooked as a colonization site for Kalotermitidae (Scheffrahn et al. 2018). In light of Emerson’s 1925 description of *Cryptotermescubioceps* from a single soldier collected from a dead liana, this host should be probed routinely as a colonization site for kalotermitids.

##### Etymology.

The species is named for the village of Ebogo II, the type locality for this termite.

**Table 3.** Measurements (mm) of *Roisinitermesebogoensis* brachypterous nymph (n=10).

Measurement	Max	Min	Mean
Head max. width	1.10	1.00	1.07
Pronotum max. width	1.16	1.08	1.11
No. antennal articles	15	15	15
Maximum diam. eye	0.20	0.20	0.20

## Discussion

Kalotermitids inhabit a single woody item and are largely unable to move to a new food source once the original is exhausted. The lone exception is *Paraneotermessimplicicornis* that builds underground galleries connecting several wood pieces (Light 1937). The ability to feed on sound wood represents a defensive adaptation in itself as the hard food source acts as an efficient physical barrier against intruders. Kalotermitids thus show low soldier-to-worker ratios (see Haverty 1977) and soldiers reach a high level of polymorphism, reflected especially in the development of the headcapsule and mandibles. Some genera such as *Bicornitermes*, *Cryptotermes*, *Eucryptotermes*, *Calcaritermes*, or *Glyptotermes*, possess very short mandibles and a plug-like headcapsule to prevent intruder entry into a nest gallery (phragmosis). In *C.cryptognathus* from Jamaica, the mandibles are reduced to small stubs that do not project beyond the frontogenal boundaries of the head capsule, and therefore cannot be used to bite opponents (Scheffrahn et al. 1998). Some other genera (e.g., *Bifiditermes*, *Epicalotermes*, *Incisitermes*, *Kalotermes*, *Neotermes*) possess long mandibles with robust dentition (crushing mandibles *sensu*Prestwich 1984) used to injure an opponent mechanically. This is often combined with release of defensive secretions originating in the labial glands (Šobotník et al. 2010, Sillam-Dussès et al. 2012). *Epicalotermespakistanicus* has particularly long and serrated mandibles (Akhtar 1974). The defensive strategy of *Roisinitermes* soldiers does not match any of these; instead, *Roisinitermes* employs a unique strategy of snapping, achieved by long and slender mandibles pressed against each other in a defensive encounter. When this potential energy is released, the left mandible springs over the right and the resultant snap is forced onto the opponent if it is in the path of the strike. This singular mandibular modification was previously known in several lineages of Termitinae (Deligne et al. 1981, Prestwich 1984, Seid et al. 2008), and was portrayed as a defensive strategy unique to this group. *Roisinitermes* represents the first undisputable evidence of parallel evolution of snapping soldiers.

Our phylogenetic analyses consistently placed *Roisinitermes* on a long branch, next to *N.insularis*. *Neotermesinsularis* is a large termite species from Northern Australia with soldiers endowed with biting mandibles of crushing type. The smaller *Roisinitermes* shares no obvious similarity with *N.insularis*, supporting its generic status. Currently, the number of mitochondrial genomes available for Kalotermitidae is limited to a handful of genera, and there is a possibility that future phylogenetic analyses will support affinities between *Roisinitermes* and yet-to-be sampled taxa. In any case, the highly unusual morphology of *Roisinitermes* suggests that it shares no close relatives among modern Kalotermitidae. Future studies should focus on whether the mechanisms used by soldiers of *Roisinitermes* to snap are like those of the distantly related Termitinae.

## Supplementary Material

XML Treatment for Roisinitermes

XML Treatment for Roisinitermes ebogoensis

## Supplementary materials

Supplementary material 1 Table S1 Data type: molecular data Explanation note: Sources and GenBank accession numbers of mitochondrial sequences used for Fig. 1. This is an open access article distributed under the terms of the Creative Commons Attribution License (CC BY 4.0), which permits unrestricted use, distribution, and reproduction in any medium, provided the original author and source are credited.Rudolf H. Scheffrahn, Thomas Bourguignon, Pierre Dieudonné Akama, David Sillam-Dussès, Jan Šobotník
